# Inoculum and pH effects on ammonium removal and microbial community dynamics in aquaponics systems

**DOI:** 10.1016/j.isci.2024.109073

**Published:** 2024-02-01

**Authors:** Peyman Derikvand, Brittany Sauter, Andrew Keddie, Lisa Y. Stein

**Affiliations:** 1 Department of Biological Sciences, University of Alberta, Edmonton, AB T6G 2E9, Canada

**Keywords:** Applied microbiology, Horticulture

## Abstract

Understanding the ecology of microorganisms is essential for optimizing aquaponics systems. Effects of pH and inoculum on ammonium removal and dynamics of microbial community composition from all compartments of lab-scale aquaponics systems were examined. Initial ammonium accumulation in systems with comammox-enriched inocula were 47% and 69% that of systems enriched with ammonia-oxidizing bacteria (AOB), with higher rates of ammonium removal and transient nitrite accumulation measured in the latter systems. By the end of operation, *Nitrosomonas* and *Nitrosospira* AOB were dominant nitrifiers in systems at pH 7.6–7.8, whereas comammox (*Nitrospira*) nitrifiers and plant growth-promoting microbes were abundant in systems operating at pH 5.8–6.0. Lower pH systems supported more robust plant growth with no significant effects on fish health. This study demonstrated functional redundancy of aquaponics microbiota, with selectivity of nitrifying taxa as a function of pH. The results suggest that inoculum and pH are important considerations for aquaponics system initiation and optimization.

## Highlights

• Comammox *(Nitrospira)* were the favored nitrifiers in lower pH aquaponics systems
• The core microbiota in aquaponics systems converged over time independent of pH
• Aquaponics systems at lower pH exhibited more robust plant growth
• Plant growth-promoting microbes were more abundant in lower pH aquaponics systems

## Graphical abstract

**Figure:**
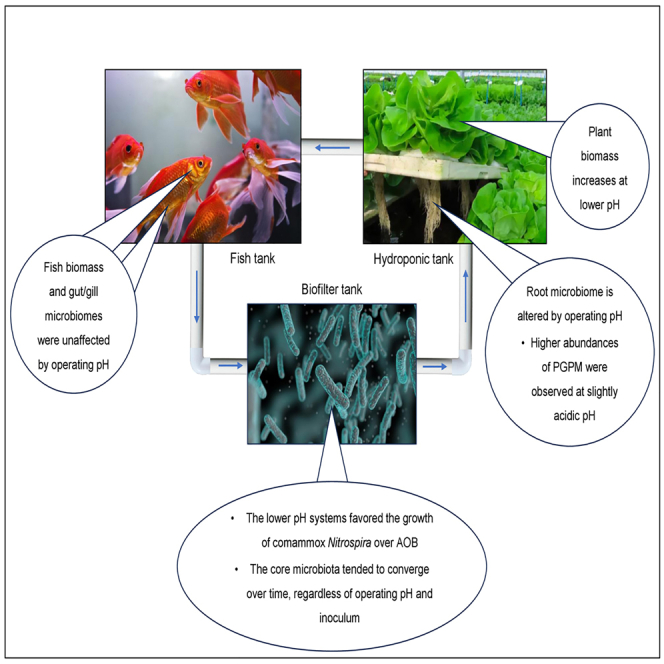

## Introduction

Manufacture and usage of artificial fertilizer, as driven by a growing world population that is shifting toward a more protein-rich diet, is severely impacting the environment. The rapid increase of global nitrogen input in agriculture, estimated at 114.6 Mt by 2024,^1^ has altered the balance of nitrogen at a global scale. Due to low nitrogen use efficiency, approximately 60% of reactive nitrogen, predominantly ammonium and nitrate, escape from soils via leaching and runoff resulting in eutrophication of freshwater and estuarine ecosystems, contamination of groundwater, and greenhouse gas emissions, mainly nitrous oxide (N_2_O).^2^^,^^3^ In addition, population growth has recently tripled the demand for aquaculture production, from 34 Mt in 1997 to 112 Mt in 2017.^4^ Aquaculture effluent contains large amounts of suspended solids, dissolved organic matter, and nitrogenous and phosphorus compounds, which require appropriate treatment prior to discharge into receiving water bodies.^5^ By combining recirculating aquaculture and hydroponics into aquaponics, nutrient-rich effluent can be used as a source of fertilizer for soilless plant cultivation, thus reducing water usage, fertilizer input, and waste discharge as compared to traditional agriculture.

Continuous water treatment to support healthy fish and crop production in aquaponics systems relies on the activity of microorganisms and their metabolic products. Nitrifiers are key microorganisms for improving water quality due to balancing reactive nitrogen pools. Nitrifiers are autotrophic microbes that oxidize ammonia released through fish gills to nitrate, which is less toxic for fish^6^ and the preferred N-source by most plants.^7^ Heterotrophic bacteria also supply micronutrients and growth-promoting molecules for plants through decomposing organic matter and solubilizing phytates, among other activities.^8^

Although heterotrophic bacteria are essential for effluent treatment in aquaponics systems, they can have negative impacts on ammonia oxidation by outcompeting the slow-growing nitrifiers. Cycling the system with low fish density or cycling with ammonium chloride amendments for 4–6 weeks before adding fish are common methods for establishing nitrifying populations in biofilters (https://pubs.nmsu.edu/_circulars/CR680/). Adjusting the water pH is also a major challenge for sustaining nitrification activity in aquaponics systems. While most plants prefer slightly acidic pH,^9^ the activity of nitrifiers decreases at pH values below 7.5 and can result in enhanced nitrous oxide emissions.^10^ Enriching the biofilter with complete ammonia-oxidizing *Nitrospira* (comammox) bacteria has been suggested to improve nitrification efficiency of aquaponics systems operating at acidic pH.^11^

Developing more profitable and environmentally sustainable food production systems using aquaponics requires a comprehensive understanding of the structure, dynamics, and activities of microbial communities involved in water treatment and promoting fish and plant health. Evaluating microbial diversity and ecology of aquaponics systems can provide a basis for managing biofilter arrangements in start-up systems and operating them toward optimum nitrogen use efficiency. Although advances have been made toward understanding the microbial ecology of aquaponics systems,^12^^,^^13^^,^^14^ little is known about the effects of pH and the initial inoculum composition on the dynamics of microbial communities over time and the complex interplay among the three biological compartments: fish, biofilters, and plants.

One aim of this study was to evaluate the effect of operating pH and the initial microbial inoculum on ammonium removal efficiency and the spatiotemporal distribution of microbial communities within and between the fish, biofilter, and plant compartments of recirculating aquaponics systems. A second aim was to compare operating pH to improve nitrogen use efficiency and plant growth in recirculating aquaponics systems.

## Results

### Ammonium removal as a function of pH and initial inoculum

Ammonium removal to nitrite and nitrate was monitored over the course of operation in four aquaponics systems: two operating at pH 7.6–7.8 (A1 and A2) and two operating at pH 5.8–6.0 (B1 and B2), each inoculated with either ammonia-oxidizing bacteria (AOB)-enriched (A1 and B1) or comammox-enriched (A2 and B2) microbial biofilters (Table 1). The ammonium concentration in all four systems increased slightly upon increasing the fish feeding rate on day 7, achieving the maximum average concentration over week two followed by varying rates of ammonium removal for each system (Table 1; Figure S1). The B1 and B2 systems reached higher maximum ammonium loads (261.1 and 180.6 μM ammonium) than the A1 and A2 systems (193.7 and 91.6 μM), respectively. Furthermore, systems with comammox-enriched inocula had 47% (A2) and 69% (B2) of the maximum ammonium load relative to systems with AOB-enriched inocula (B1 and B2, respectively). Systems B1 and B2 (pH 5.8–6.0) showed slower ammonium removal rates and required more time to achieve stability compared to systems A1 and A2, respectively. A significant difference was observed among the four systems (Kruskal-Wallis, p value = 0.046, eta2[H] = 0.41), although Dunn’s test indicated that only system A1 had significantly higher ammonium removal rates than the other three systems by the end of operation (p values: A1 to A2 = 0.0002, A1 to B1 = 0.001, A1 to B2 = 0.0001, A2 to B1 = 0.43, A2 to B2 = 0.87, B1 to B2 = 0.27). A slight rise in ammonium concentration after lettuce harvesting and transplanting of new seedlings at day 28 and day 56 was observed in all four systems (Figure S1).

**Table 1:** Average ammonium removal rates and percentage of ammonium removal for each week of operation in the four aquaponics systems

Week of operation	Average ammonium removal rates (μM per day)							
	System A1		System A2		System B1		System B2	
	Avg. rate	% Removal	Avg. rate	% Removal	Avg. rate	% Removal	Avg. rate	% Removal
*2 Max. Conc.*	*193.7*		*91.6*		*261.1*		*180.6*	
3	−9.0	−5	7.1	8	31.6	12	31.7	18
4	56.7	29	48.9	53	49.1	19	55.7	31
5	86	44	51.6	56	93.6	36	70.9	39
6	97.6	50	55	60	116.4	45	86.2	48
7	125.3	65	55.8	61	130.2	50	95.4	53
8	140	72	59.3	65	161.3	62	95.3	53
9	144	74	58.6	64	173.1	66	106.9	59
10	135.9	70	55	60	169.1	65	74.6	41
11	136.2	70	58.1	63	188.2	72	119.1	66
12	168.7	87	60.3	66	179.3	69	124.9	69
13	170.7	88	68.3	75	204.1	78	133.6	74
14	168	87∗	67	73	198.5	76	129.6	72

The maximum average ammonium concentration was measured over week 2 and is assumed to represent the minimum concentration of ammonium released by the fish each day for each system. The average rates of ammonium removal per day were determined by subtracting the average concentrations of ammonium over each week of operation from the maximum average concentration (measured over week 2) divided by 7 days. The % removal was calculated as the average rate of removal for each week divided by the average maximum ammonium concentration. Ammonium removal rates by system A1 (∗) were significantly higher than the other three systems by the end of operation (p < 0.05).

Nitrite accumulation was not observed for systems B1 or B2, while in systems A1 and A2 initial nitrite levels were substantial (ca. 5 μM), but rapidly declined to ca. 0.5 μM within 7 days of operation (Figure S2). Nitrate concentrations increased from over the second week of operation due to the increased fish feeding rate (Figure S3). However, nitrate was lower in the B1 and B2 systems compared to the A1 and A2 systems until the microbial communities were established after 9 weeks, which coincided with the third round of lettuce cultivation. No significant difference in nitrate levels was observed among the four systems (Kruskal-Wallis, p value = 0.12). Similar to ammonium levels, nitrate levels also spiked between lettuce harvest and transplant of new seedlings, as uptake by the plants was disrupted during this interval. As with ammonium and nitrite, levels of nitrate remained unchanged up to 5 h following fish feeding (Figures S1–S3).

### Biofilter microbiome communities converge over time and sustain both AOB and comammox populations

**Figure 1:**
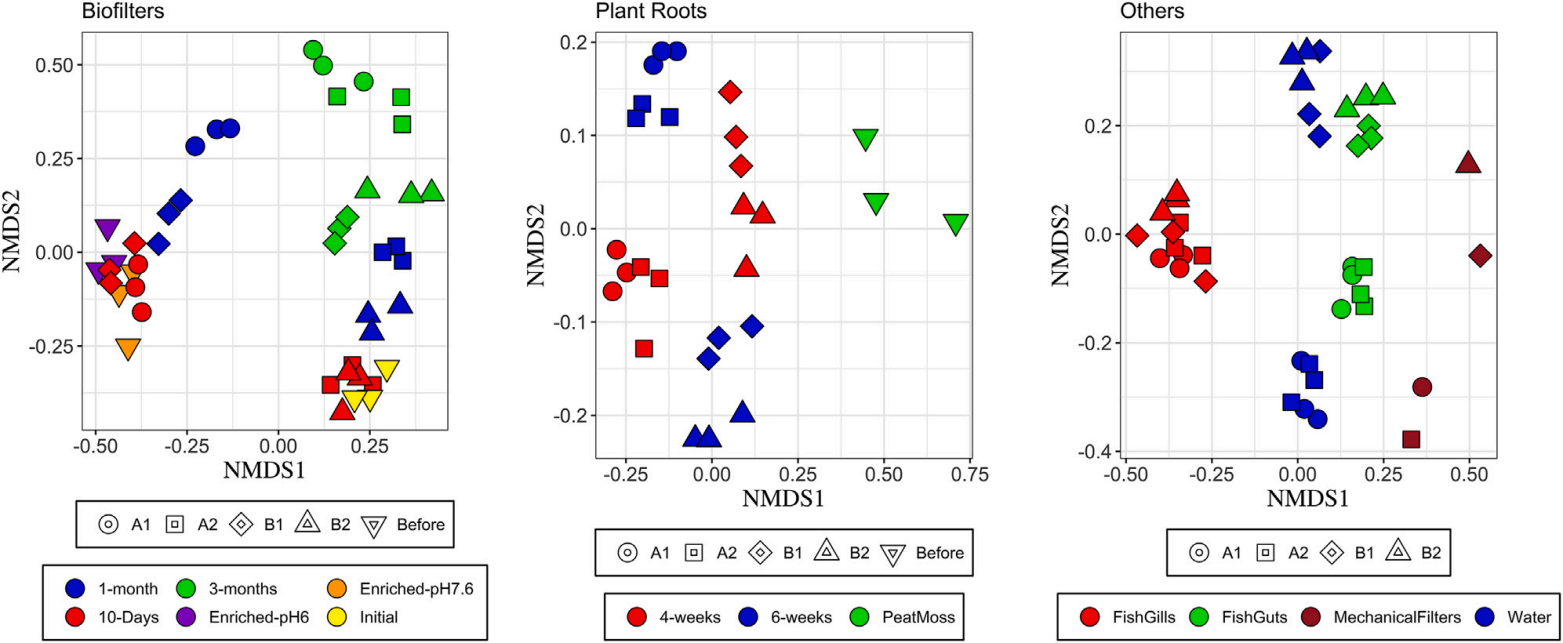
Nonmetric multidimensional scaling ordination (Bray-Curtis dissimilarity) representing the grouping of samples per compartment Shapes represent aquaponics systems. Shifts in the beta-diversity of biofilters and plant roots were investigated over time, and colors show different time intervals. Fish guts/gills, water samples, and mechanical filters were investigated at the end of operation.

**Figure 2:**
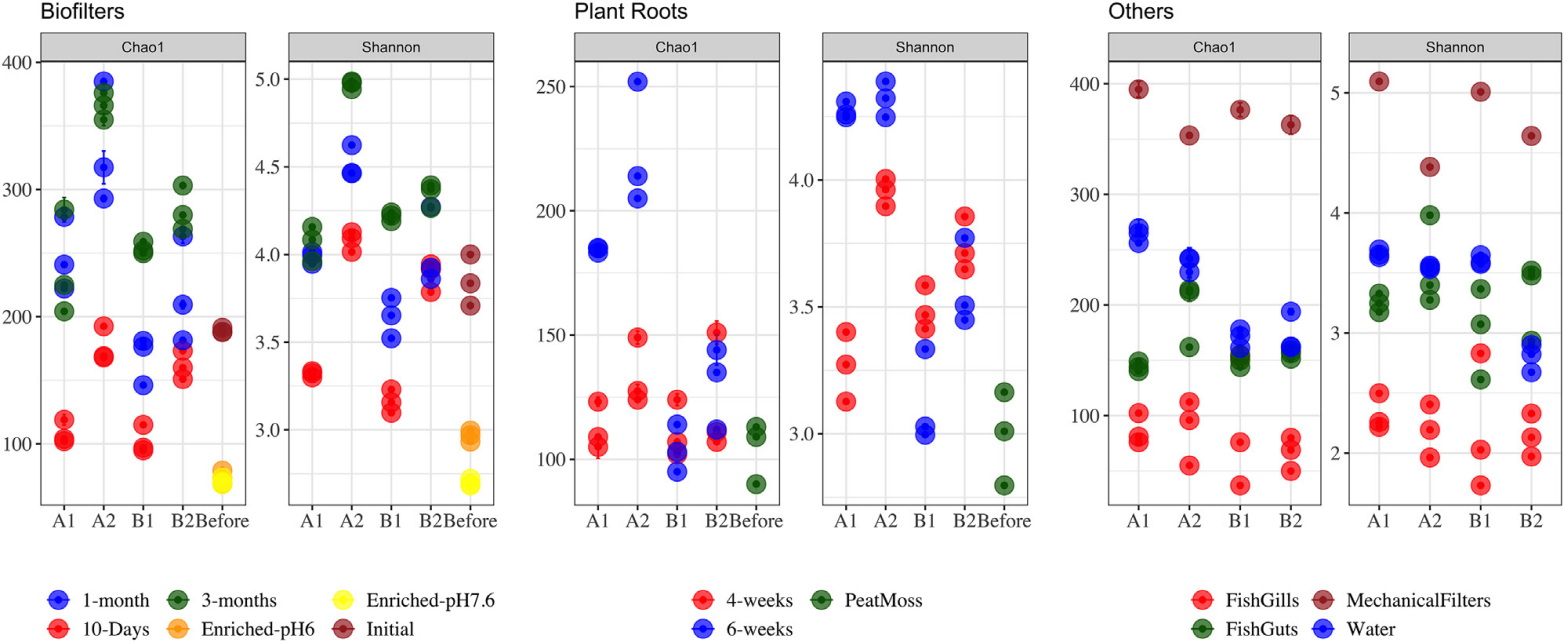
Richness (Chao1) and diversity (Shannon) distribution for recovered ASVs of different aquaponics compartments Shifts in the alpha-diversity of biofilters and plant roots were investigated over time, and colors show different time intervals. Fish guts/gills, water samples, and mechanical filters were investigated at the end of operation.

To study the dynamics of biofilter microbial communities over time, amplicon sequence variant (ASV) diversity was compared after 10, 30, and 90 days of aquaponics operation. Non-metric multidimensional scaling (NMDS) revealed that the microbial composition among the biofilm carriers was significantly different depending on the sampling date (Permanova, p = 0.001) (Figure 1). Pairwise permutational ANOVA (PERMANOVA) test showed significant differences between biofilters from systems with AOB-enriched (A1 and B1) versus comammox-enriched inoculum (A2 and B2) at 10 days of operation (p = 0.015). These differences decreased over time, with higher similarity among all four biofilter communities after 90 days of operation. Biofilters from systems operating at the two pH values (A1/B1 versus A2/B2) also showed greater similarity to one another after 3 months of operation (Figure 1). Both richness (Chao1) and diversity (Shannon) of the biofilter samples increased significantly over time for all biofilter communities (ANOVA, p < 0.05); however, the highest richness and diversity was measured for communities from system A2 (Figure 2).

After 3 months of operation, the proportion of Actinobacteria and Chloroflexi was higher in biofilter communities from systems B1 and B2 than systems A1 and A2. (Figure S4). The abundance Planctomycetota increased over time in systems A2, B1, and B2, which might be attributed to growth of *Pirellulaceae* spp. (Table 2), whereas Cyanobacteria increased in abundance only in system A1. The proportion of Proteobacteria populations from systems A2 and B2 were generally more stable than those from systems A1 and B1, which declined from day 10 to 3 months. ASVs related to the Hydrogenedentes phylum were abundant in system B1 at day 10 and 1 month, but mostly disappeared by 3 months.

**Table 2:** Relative abundances (%) of the dominate microbial families from amplicon sequences determined in compartments of bench-scale aquaponics systems operating at pH 7.5–7.8 (A1 and A2) or pH 5.8–6.0 (B1 and B2) inoculated with unenriched (A1 and B1) or comammox-enriched (A2 and B2) biofilters

Family	% ASV Groups: Biofilters											
	A1			A2			B1			B2		
	10 days	1 month	3 months	10 days	1 month	3 months	10 days	1 month	3 months	10 days	1 month	3 months
Nitrosomonadaceae	4.2	2.2	2.3	16.8	2.3	2.8	3.1	2.5	0.4	0.1	0.4	0.5
Nitrospiraceae	0.5	5.4	1.6	1.9	5.9	3	0.3	2	1.3	1.1	2.1	4.7
Nitrososphaeraceae	0.03	0.03	0.01	0.1	0.06	0.04	0.01	0.08	0.05	0.2	0.07	0.06
Mycobacteriaceae	0	0.4	4.4	0.4	0.9	2.6	0.6	3.3	8.8	0.7	1.4	5.7
Caldilineaceae	0.02	5.2	2	0.3	0.9	2.3	0	0.2	1.2	0.3	0.8	3.2
Flavobacteriaceae	0	0.01	1.5	0	0.04	1.4	0	0.02	0.2	0	0	0.1
Comamonadaceae	1	1.9	4.8	1.4	1.6	2.1	2.1	1.9	3.7	1.4	0.9	2.6
Aeromonadaceae	0	3.2	7.8	0	0.3	3.9	0	0	0	0	0	0.4
Pirellulaceae	1.7	3.9	6.6	3	5.3	7.8	1.9	3.1	9.6	2.8	4.5	8.7
Pseudomonadaceae	0	0.9	6.7	0.1	0.8	2	0	0	0.09	0.01	0.04	0.05
Nocardiaceae	0.2	1.1	1.6	0	0.4	1.5	0.03	0.3	0.2	0	0.2	0.3

	% ASV Groups: Plant roots									
	Peat moss	A1		A2		B1		B2		
		4 weeks	6 weeks	4 weeks	6 weeks	4 weeks	6 weeks	4 weeks	6 weeks	6 weeks
Mycobacteriaceae	3.4	0.5	1.8	0.4	2	2	8.3	2.9	10.7	3.1
Micromonosporaceae	3.6	0.1	4.9	0.1	3.8	3.8	0.2	35	0.4	29
Pirellulaceae	0	2.7	19.3	0.9	21.9	21.9	2.1	6.6	1.5	6.2
Gemmataceae	0.1	7.9	11.4	13	11.2	11.2	15.6	9.9	12.9	8.9
Aeromonadaceae	0	0.8	1.1	0.1	0.9	0.9	0.1	0.4	0.2	0.2
Nitrospiraceae	0	0.4	2.1	0.9	1.9	1.9	0.1	0.2	0.1	0.1
Roseiflexaceae	0	0.1	5.2	1.8	4.1	4.1	0.7	6.4	1.5	9.2
Microbacteriaceae	10.6	0.1	0.4	0.1	0.3	0.3	4.8	2.1	3.4	1.4
Rhodobacteriaceae	5.1	4.4	5.3	6.1	4.5	4.5	4.3	4.4	4.2	3.9
Ilumatobacteriaceae	0	1.3	4.6	1.1	4.1	4.1	0.6	0.3	0.2	0.4
Rubritaleaceae	0	4.3	3.9	3.1	3	3	0	0.09	0	0
Comamonadaceae	0.5	6.1	5.1	7.2	5.4	5.4	3.1	3.7	5.2	4.8
Verrucomicrobiaceae	2.2	1.6	2.3	1.9	1.7	1.7	0	0	0	0.06
Beijerinckiaceae	0	0	1.2	0.1	1.1	1.1	0.1	1.9	0.4	1.2
Frankiaceae	8.1	0	0	0	0	0	0	0	0	0

	% ASV Groups: Fish guts				% ASV Groups: Fish gills			
	A1	A2	B1	B2	A1	A2	B1	B2
Pseudomonadaceae	0.1	0.4	0.09	0.1	43	58	50	54
Weeksellaceae	0	0	0	0	29	26	31	25
Caulobacteraceae	0	0	0	0	10.8	7.5	7.1	9.4
Comamonadaceae	0.1	0.1	0.1	0	6.3	2.9	3.7	5.5
Moraxellaceae	0	0.1	0	0.1	2.2	1.2	3.1	1.7
Nitrospiraceae	0	0	0	0	1.1	0.6	0.6	0.7
Nostocaceae	40	32	38	19	0.2	0.8	0.4	0.2
Mycobacteriaceae	23	11	20	39	0.2	0.4	0.4	0.3
Rhodobacteraceae	4.2	6.1	5.5	5.9	0.3	0.2	0.2	0.3
Nocardiaceae	1.9	7.9	1.8	2	0.2	0.2	0.4	0.1
Gemmataceae	5.6	5.9	5.6	4.4	0	0	0	0
Pirellulaceae	3.4	6.1	3.7	3.9	0.07	0.05	0.2	0
Isosphaeraceae	3	2.7	2.1	5.6	0	0		0

Among the nitrifier populations, AOB in the Nitrosomonadaceae, including the genera *Nitrosomonas* and *Nitrosospira*, made up 4.2% (A1), 16.8% (A2), 3.1% (B1), and 0.1% (B2) of the ASVs at day 10, and reached 2.3% (A1), 2.8% (A2), 0.4% (B1), and 0.5% (B2) of ASVs after 3 months of operation (Table 2). ASVs related to *Nitrospira* represented 0.5% (A1), 1.9% (A2), 0.3% (B1), and 1.1% (B2) at day 10 and increased to 1.6% (A1), 3% (A2), 1.3% (B1), and 4.7% (B2) by 3 months of operation (Table 2).

**Figure 3:**
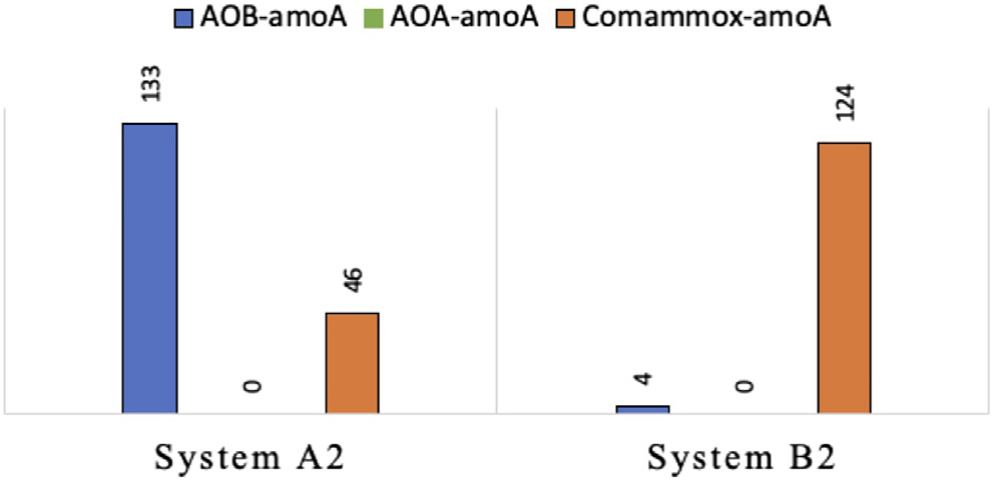
HiFi reads assigned to AOA, AOB, and comammox *amoA* genes in metagenomes of biocarrier samples from systems A2 and B2 PacBio metagenomic analysis was conducted on DNA samples collected at the end of the operation period.

Metagenomic analysis of biocarrier samples confirmed that 74% of the ammonia monooxygenase subunit A (*amoA*) genes in system A2 belonged to AOB species and 26% belonged to comammox *Nitrospira* bacteria, while in system B2 comammox *Nitrospira amoA* was dominant (97% of *amoA* gene sequences) and only 3% of the *amoA* gene sequences were assigned to AOB (Figure 3). Similarly, consistently low quantities (<1%) of ASVs in the amplicon libraries and no *amoA* genes in the metagenomes related to ammonia-oxidizing archaea were detected across all four systems, indicating negligible contributions to nitrification activity from this microbial group.

### Plant biomass increases at lower pH and root microbiome is altered by operating pH

Three rounds of lettuce were cultivated in each of the four aquaponics systems to compare the effect of microbial inoculum and operating pH on plant biomass and root microbiota. Lettuce was harvested after 4 weeks of growth in the first and second rounds and after 6 weeks of growth in the third round. No relationship was found between microbial inoculum and effects on final plant biomass, but consistently higher biomass was achieved in systems B1 and B2 (pH 5.8–6.0) versus systems A1 and A2 (pH 7.6–7.8) for each round of lettuce growth, although the combined difference over the three rounds was not significant (ANOVA, = 0.11, eta2 = 0.08), likely due to the two weeks of additional growing time for lettuce in round three (Figure S5).

NMDS plots showed that rhizosphere microorganisms sampled after 4 weeks or 6 weeks of lettuce growth clustered into distinct groups relative to seedlings in peat moss (pairwise PERMANOVA, p = 0.023), indicating that time was an important factor for microbiome development (Figure 1). Comparisons between systems A1 and A2 with B1 and B2 revealed a significant impact of operating pH on root microbiota composition (pairwise PERMANOVA, p = 0.011). Based on the Chao1 index, richness of the root microbiota in systems A1 and A2 significantly increased over time, but not in systems B1 and B2 (Figure 2). Conversely, the Shannon index measurements indicated a significant increase in the diversity of root microorganisms over time at both pH levels. Development of root microbiota was not significantly influenced by the source of the initial inoculum, suggesting that the rhizosphere microbiome developed independently from the biofilter communities.

In terms of community composition, the two major ASV phyla observed in the lettuce rhizosphere from seedlings growing in peat moss were Actinobacteriota (31%) composed of the families Microbacteriaceae (10.6%) and Frankiaceae (8.1%), and Proteobacteria (64%) (Figure S6; Table 2). Lower pH favored the growth of Actinobacteriota, comprising 43% of ASVs from the 6 weeks lettuce rhizosphere in systems B1 and B2 (Figure S6; Table 2). Differential abundance analysis indicated that the quantity of Microbacteriaceae (Ancom-BC2, lfc = 4.08, p = 0.00001), Mycobacteriaceae (lfc = 2.31, p = 0.00110), Kineosporiaceae (lfc = 3.64, p = 0.00002), and Micromonosporaceae (lfc = 4.01, p = 0.00001) was significantly higher in the rhizosphere samples of the slightly acidic systems compared to systems A1 and A2 (Table S2). In contrast, higher pH favored the growth of Nitrospiraceae (lfc = 3.92, p = 0.00001), Verrucomicrobiaceae (lfc = 3.56, p = 0.00003), and Vicinamibacteraceae (lfc = 2.81, p = 0.00022) on the plant roots.

Interestingly, nitrogen fixing Frankiaceae were absent from the rhizosphere of all systems, likely due to the ready availability of nitrate (Table 2). Planctomycetota, mainly Gemmataceae, were higher in systems A1 and A2 than systems B1 and B2, increasing to 29% of ASVs in the 6 weeks lettuce rhizosphere, but the difference was not statistically significant. Proteobacteria were abundant at 29% and 25% in the 4 weeks and 6 weeks lettuce rhizosphere, respectively.

### Fish biomass and gut/gill microbiomes were unaffected by operating pH

The four aquaponics systems were stocked with five goldfish per tank with initial weights between 20 and 25 g/fish. No fish mortality was observed for the duration of the experiment. Fish biomass in systems A1 and A2 reached 31.9 and 31.4 g, respectively, slightly higher than in the B1 (23.7 g)and B2 (27.4 g) systems, although not statistically significant (Kruskal-Wallis test, p = 0.15, eta2[H] = 0.04) (Figure S7).

NMDS plots showed that microorganisms collected from water, fish feces (collected in mechanical filters), and fish digestive systems (guts) in the A1 and A2 versus the B1 and B2 tanks clustered into distinct groups (Figure 1). Despite these differences, the microbial inoculum source did not influence either water or fish-associated microbial populations. The diversity of microorganisms from water, fish gills, and fish guts was similar in all systems based on the Shannon index, whereas the Chao1 index indicated that the A1 and A2 microbiomes had higher species richness than the B1 and B2 microbiomes (Tukey, p < 0.05; Figure 2).

The major microbial phyla of ASVs in fish guts were Cyanobacteria (37%), Actinobacteria (34%), Proteobacteria (17%), and Planctomycetota (13%) (Figure S8). These same phyla with ASV relative abundances of 1.5%, 10%, 16%, and 20%, respectively, were detected in the mechanical filters collecting fish feces (Figure S9). Mechanical filters were dominated by ASVs related to Firmicutes (31%) but also contained ASVs related to *Nitrospira* (2.7%). Compared to fish intestines, a higher diversity of phylum-level ASVs was observed in the mechanical filters, which shared similar phyla with the bulk water samples (Figure S10). High abundances of ASVs related to Bacteroidota and Proteobacteria were found in both water and fish gill samples (Figures S10 and S11), but with higher relative abundances in water samples at pH 5.8–6.0 than at pH 7.6–7.8. Also, Actinobacteria dominated ASVs in the pH 5.8–6.0 (54%) compared to pH 7.6–7.8 water samples (10.5%). A low percentage of ASVs related to *Nitrospira* were detected in all fish gills (Table 2). Unlike the biofilter and rhizosphere microbial communities, the diversity and composition of the microbiome of fish guts/gills were not influenced by operating pH.

### The majority of ASVs belong to their independent niche in aquaponics systems

**Figure 4:**
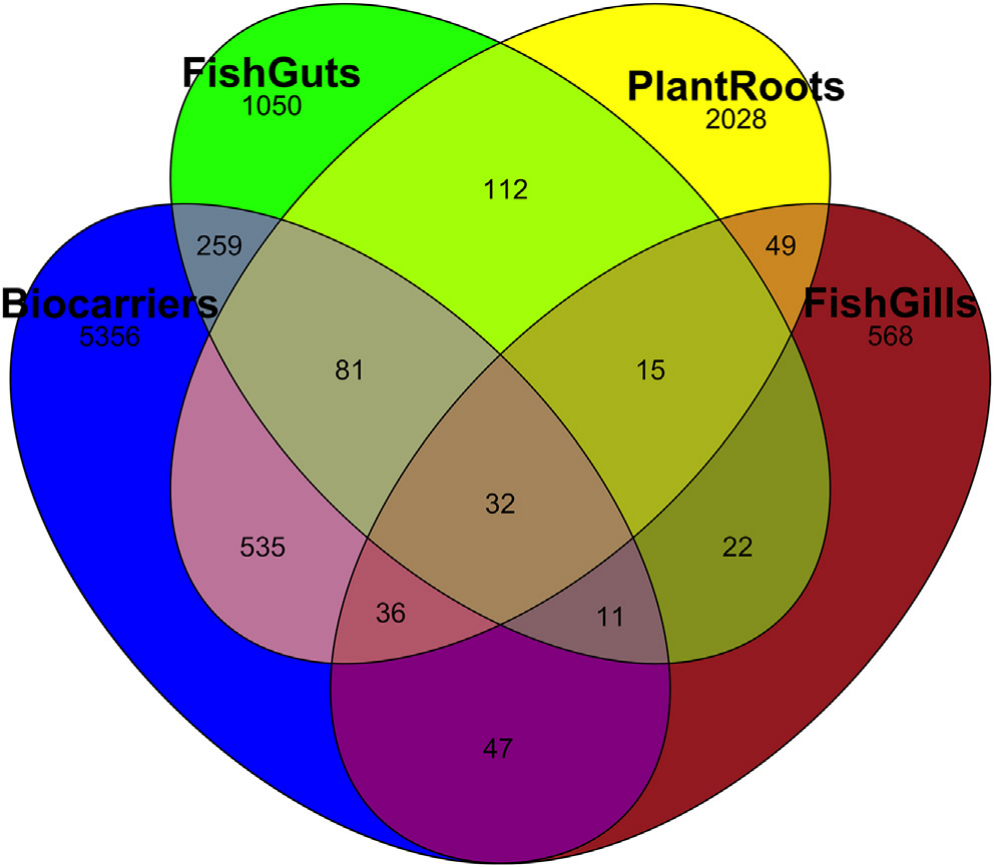
Venn diagram representing the proportion of common and unique ASVs in different biological compartments of aquaponics The highest number of ASVs were shared by biocarriers and plant roots. Fish intestines and fish gills were discovered to share the slightest similarities.

Plotting the distribution of ASVs across all samples provided evidence for unique and shared microbial groups between and among the aquaponics compartments (Figure 4). The vast majority of identified ASVs were specific to each individual compartment. However, the core microbiome detected among all three biological compartments, including fish gills/guts, lettuce rhizosphere, and microbial biofilters, were mainly attributed to the genera *Mycobacterium*, *Nocardia*, *Pirellula*, and *Bacillus*. 88% of the microbiota were unique for each compartment, with 52.5%, 19.8%, 10.2%, and 5.5% belonging to biocarriers, plant roots, fish guts, and fish gills, respectively. Biocarriers shared 684, 383, and 126 ASVs with plant roots, fish gills, and fish guts, respectively. The smallest number of shared ASVs was between fish gills and guts and was in the *Pseudomonas*, *Aeromonas*, *Acinetobacter*, and *Serratia* genera.

## Discussion

The aim of this study was to investigate how the source of microbial inocula and operational pH influenced both nitrogen removal rates and microbial community composition in the compartments of an aquaponics system. Nitrogen (ammonium, nitrite, and nitrate) measurements in the fish tanks revealed that systems A2 and B2 inoculated with comammox-enriched biocarriers achieved a smaller maximum concentration of ammonium but were also slower to remove ammonium relative to systems A1 and B1 that were inoculated with AOB-enriched biocarriers. The benefits of initiating aquaponics systems with microbial communities enriched with nitrifiers have been reported in other studies.^15^^,^^16^ Thus, selection of a nitrifier-enriched inoculum, whether by comammox or AOB, can be a useful strategy to enable desired start-up conditions and stabilization time for aquaponics systems rather than operating at low fish density or amending with ammonium chloride for several weeks (Sallevane, 2016). In terms of operating pH, lower quantities of nitrate were measured in systems B1 and B2 (pH 5.8–6), which was likely due to the higher rate of plant growth. These results were similar to those of Wongkiew et al.^17^ where lower nitrate and higher ammonium concentrations were measured in a system operating at pH 6 versus pH 7. The quantity of nitrite and nitrate represents an equilibrium between nitrification activity and plant absorption, which also explains the temporary increases in ammonium and nitrate concentrations observed between plant harvest and re-planting (Figure S3).

Systems B1 and B2 (pH 5.8–6.0) consistently yielded higher lettuce biomass, likely due to the optimized pH for plant nutrient uptake.^18^ The positive effect of lower pH on lettuce growth was less pronounced in the 6-week-old plants (29% difference) compared to the 4-week-old plants (58% and 53% differences), which is consistent with the study by Tyson et al.^19^ where lower pH had a greater effect on early crop yield than on later yields. In contrast, slightly lower fish biomass was obtained in the B1 and B2 systems, perhaps due to the higher ammonium concentrations^20^ or the negative impact of acidic pH on fish growth rates.^21^ Despite the difference in fish growth, no mortality or negative behavior was observed.

This study validated that pH remains a strong determinant of microbial community composition in aquaponics bio-compartments.^10^^,^^17^ Comparisons of beta diversity and microbial composition associated with biofilm carriers at two pH levels showed convergence from initial populations to those after 3 months of operation. Interestingly, nitrifiers in the genera *Nitrosomonas* and *Nitrosospira* made up 2%–3% of the biofilter microbiota at pH 7.6–7.8 at the end of operation, which is similar to other aquaponics systems,^17^^,^^22^ whereas the biofilter microbiota operating at pH 5.8–6.0 were mainly *Nitrospira* (1.3%–4.7%) at the end of operation. Despite the lower nitrification rate of comammox *Nitrospira*, their presence could be advantageous for reducing nitrite accumulation in the water^23^ and lowering the emission of nitrous oxide.^24^ Nitrification by comammox *Nitrospira* has been reported in highly efficient aquaponics systems, similar to what is reported here.^13^^,^^14^ Nevertheless, ASVs and metagenome signatures of comammox *Nitrospira* were present in the biofilter communities after 3 months of operation. Ammonia-oxidizing archaea, however, were not a major population in any of the systems.

Higher abundances of ASVs related to *Actinobacteria*, characterized as plant growth-promoting microbes (PGPM), were observed in the microbial biofilters, water, and lettuce rhizosphere samples in the pH 5.8–6.0 systems. Members of this phylum including strains of *Mycobacterium*,^25^ *Nocardia*,^26^ *Leifsonia*,^27^ and *Sporichthya*^28^ were identified. These microbes can significantly promote nutrient uptake and plant growth. Water samples from the systems at pH 5.8–6.0 especially favored the presence of ASVs related to the endophytic actinobacterial genera *Micromonospora* and *Actinoplanes* (Micromonosporaceae family), also known to enhance plant growth.^29^ *Rhizobacteria* appeared to thrive at the lower pH, which also correlated with more robust plant growth. Upon transferring lettuce seedlings to the hydroponics grow beds, ASVs related to *Frankia* bacteria disappeared, and *Gemmata* and *Pirellula* ASVs appeared, which could be in correlation with increased flavonoid content of the plants.^30^ Furthermore, ASVs related to *Bacillus*, *Paenibacillus*, and the Proteobacteria *Rhodobacter* and *Pseudomonas* were present in all rhizosphere samples, including those from peat moss. Some strains of these genera are among the most widely reported PGPM that stimulate plant growth mainly through solubilizing phosphate and producing siderophores.^31^^,^^32^

Compared to biofilters and rhizospheres, low phylogenetic diversities and similar ASV groups were observed in the fish gills and guts at both pH levels. The major fish-associated bacterial phyla were consistent with previous studies^33^^,^^34^ reporting the presence of Proteobacteria, Cyanobacteria, Planctomycetes, and Actinobacteria as core microbiota of carp species. Some aquaponics studies sampled fish feces to represent fish gut microbiota.^14^^,^^16^ Our results showed that fish feces microbiota resembled the water microbiota. Similar to fish guts, low phylogenetic diversity among ASVs were identified in the fish gill samples. Despite direct contact with water, the microbiomes of fish gills were not affected by the operating pH. Similar phyla with the same relative abundances in all fish gills samples indicate that these microorganisms likely contribute to gill function. Low quantities of *Nitrospira* ASVs found in fish gills may benefit from ammonium supplied by the host. Bacterial ammonia oxidation and the presence of nitrifying microbes (AOB) in fish gills have been reported previously.^35^

### Conclusions

Aquaponics are constructed ecosystems designed for the efficient farming of fish and crops. The three biotic compartments of these systems—fish, plants, and microorganisms—are connected and dependent on one another through the recirculation of water and flow of nutrients. Along with researching aquaponics microorganisms to enhance nitrogen use efficiency, mesocosm studies provide a tool for examining the potential influence of environmental factors, like pH and microbial inocula, on functional groups of microorganisms like nitrifiers and PGPM in larger scale systems. This study showed that despite differences in the abundance of particular phyla, the core microbiota tended to converge, regardless of operating pH and source of inoculum, although pH was selective for AOB or comammox *Nitrospira*. The results suggest that groups of microorganisms with defined functions are supported by aquaponics operations that are selected for and maintained within a range of operating conditions. This study also showed that while both comammox and AOB nitrifiers can be sustained in aquaponics systems, the lower pH systems favored comammox over AOB. Thus, decreasing the pH in systems with low abundances of comammox *Nitrospira* could result in transient accumulation of ammonia until the population can grow and stabilize.

### Limitations of the study

This study used aquaponics mini-systems to investigate the effect of pH on microbial communities, and fish and plant growth. Nevertheless, bench-scale systems do not substitute for performing similar studies in active commercial-scale operations to compare the ammonium removal and microbiome composition dynamics at scale. In addition, conducting studies with different combinations of fish and plants is needed to gain a thorough comprehension of the organization, behavior, and functions of microbial communities responsible for water treatment and supporting the well-being of fish and plants.

Operating at a lower pH altered the composition of microbial communities in fish guts, lettuce rhizospheres, and the heterotrophic bacteria associated with the biofilters and resulted in increased plant growth. Further investigations using metagenomic tools are required to obtain valuable insights regarding other functions of important microbes like those involved in iron and phosphorous availability, other N-cycle processes, the presence of pathogens, and detailed contributions of PGPM to plant productivity in aquaponics systems.

## STAR★Methods

### Key resources table

**Table:** 

REAGENT or RESOURCE	SOURCE	IDENTIFIER
**Biological samples**		

Lactuca sativa	Mckenzie Seeds Company	Cat#140123
Carassius auratus	Department of Biological Sciences, University of Alberta	N/A
Microbial communities from aquaponic’s biofilter tank	NutraPonics Inc. Alberta, Canada	N/A

**Chemicals, peptides, and recombinant proteins**		

Sodium nitroprusside	Fisher Scientific	Cat#735536
Sodium hydroxide	Fisher Scientific	Cat#1310
Sodium dichloroisocyanurate	Sigma-Aldrich	Cat#2893
Ammonium sulfate	Sigma-Aldrich	Cat#7783
Sulfanilamide	Fisher Chemical	Cat#63741
Naphthylethylene diamine dichloride	Fisher Scientic	Cat#721276
Potassium nitrate	Thermo Scientific	Cat#013443
Copper sulfate	Sigma-Aldrich	Cat#MKCM5523
Zinc sulfate	Acros Organics	Cat#A0312675
Hydrazine sulfate	Sigma-Aldrich	Cat#BCCC2163
Sodium nitrite	Sigma-Aldrich	Cat#7632

**Critical commercial assays**		

FastDNA Spin Kit for Soil	MP Biomedicals Inc.	Cat#116560
Fresh Water Master Test Kit	API	Cat#01034

**Deposited data**		

16S rRNA amplicons sequencing data	NCBI SRA database	NCBI: PRJNA941956
metagenomic sequencing data	NCBI SRA database	NCBI: PRJNA1022846

**Oligonucleotides**		

16S-341F primer	Genome Quebec	CCTACGGGNGGCWGCAG
16S-805R primer	Genome Quebec	GACTACHVGGGTATCTAATCC

**Software and algorithms**		

R programming language	https://cran.r-project.org/bin/macosx/	R4.2.1
DADA2 pipeline	https://benjjneb.github.io/dada2/tutorial.html	1.16
package ‘vegan’	https://github.com/vegandevs/vegan	N/A
package ‘ggplot2’	https://github.com/tidyverse/ggplot2	N/A
package ‘phyloseq’	https://joey711.github.io/phyloseq/	N/A
Taxonomic-Profiling-Diamond-Megan pipeline	https://github.com/PacificBiosciences/pb-metagenomics-tools/tree/master/Taxonomic-Profiling-Diamond-Megan	N/A
MEGAN6 toolbox	https://uni-tuebingen.de/fakultaeten/mathematisch-naturwissenschaftliche-fakultaet/fachbereiche/informatik/lehrstuehle/algorithms-in-bioinformatics/software/megan6/	6_25_3

### Resource availability

#### Lead contact

Further information and requests for resources should be directed to and will be fulfilled by the Lead Contact, Lisa Y. Stein (lisa.stein@ualberta.ca). The dataset is not declared to be publicly accessible.

#### Materials availability

This study did not generate new unique reagents.

#### Data and code availability

• The 16S rRNA amplicon sequencing data generated in this study have been deposited in the NCBI SRA database (accession number: PRJNA941956). The metagenomic sequencing data have been deposited in the NCBI SRA database (accession number: PRJNA1022846). These data are publicly available.
• All relevant data supporting the findings of this study are available from the lead contact upon request.
• The published article and supplemental information include all data generated and analyzed during this study. This paper does not report original codes.

### Experimental model and study participant details

#### Ethics statement, animal subjects

This research was conducted in accordance with institutional standards for animal care and use by The University Animal Policy and Welfare Committee (UAPWC), University of Alberta, Canada.

#### Aquaponics microcosms

Four floating raft aquaponics bench-scale systems were built and maintained in the Department of Biological Sciences, University of Alberta. Each bench-scale system consisted of a recirculating aquaculture unit (fish tank and biofilter tank) and a hydroponic bed above the system with a total of 320 L volume and 250 L water volume (based on original designs of Dr. Nick Savidov, Lethbridge College, Alberta, Canada). Water from the fish tanks flowed to the biofilter tanks, and then to the hydroponic beds using submerged circulation pumps with a flow rate of 10 L/min, before returning to the fish tanks. Water loss due to evaporation and evapotranspiration was compensated by replenishing the systems daily with dechlorinated water. Suspended solids were continuously removed from fish tanks using mechanical filters (AquaClear 70 Power Filter). Air was supplied to the fish tanks and grow beds to a dissolved oxygen (DO) concentration of ∼7 mg/L. Each hydroponic tank contained a single raft supporting 14 plants equipped with overhead LED lighting (Fluence RAZRx LED Grow Light System) operating 18 h/d. Tanks were covered with corrugated plastic sheets to prevent algal growth.

Four aquaponics systems were operated side by side in a temperature-controlled room (23°C) for 3.5 months. Two systems were maintained at pH 7.6–7.8 (A1 and A2) and two were maintained at pH 5.8–6.0 (B1 and B2). Two systems were inoculated with microbial biofilm carriers (Kaldnes, Veolia Water Technol., Sweden) derived from a commercial scale aquaponics system (NutraPonics Inc. Alberta, Canada, curtosy of Dr. Nick Savidov, Lethbridge College, Alberta, Canada; A1 and B1) and two were inoculated with microbial biofilm carriers previously adapted to pH 5.8^11^ (A2 and B2). Each system was stocked with five goldfish with initial weights of 20–25 g/fish approximating a density of 1 kg/m^3^. Fish were fed 1 g floating fish pellets (Mazuri Bits) per day per tank for the first week, increasing to 4 g twice per day at 9 a.m. and 5 p.m. for the remainder of operation. The total fish biomass in each system was calculated by subtracting the initial from the final fish weight at the end of the operation. Differences in the fish biomass between the four systems were compared using Kruskal-Wallis test and eta squared based on the H-statistic was conducted in r to calculate the effect size. Lettuce seeds were germinated in peat moss two weeks prior to transplant into the grow beds. Three rounds of crops were grown and harvested for biomass measurements: the first and second rounds after 4 weeks of growth and the third round after 6 weeks of growth. ANOVA test was used to detect significant differences in lettuce biomass among the four systems, and Eta squared was used to measure the effect size.

### Methods details

#### Measurement of ammonium removal capacity

Water samples (n = 3) were collected from the fish tanks 30 min, 2 h and 5 h after fish feeding every other day over the course of the operation. Ammonium, nitrite, and total nitrite + nitrate were measured using colorimetric assays in 48-well microplates (Multiskan Spectrum, Thermo Fisher Scientific).^11^ Differences in ammonium concentrations between the four systems, the point of highest ammonium load, were compared during the second week of operation using the Kruskal-Wallis test and Dunn’s multiple comparison test. Changes in the ammonium removal rate per day from week to week was calculated by subtracting the average ammonium concentration measured over each week from the average ammonium concentration measured at week 2 for each system divided by 7. These measurements assume that the fish minimally produced the same average amount of total ammonium measured for each system during week 2 throughout the remainder of the operation time. A Dunn’s multiple comparison test was used to detect significant differences in the percentage of ammonium removal among the systems during the last week of operation.

#### Sample collection and DNA extraction

Biofilm carriers were collected from biofilter tanks after 10 days, one month, and three months of operation. Five biofilm carriers from each of the four systems were separately added to sterile 50 mL falcon tubes containing 10 mL sterile Milli-Q water. Tubes were vortexed vigorously for 5 min to detach biofilm from the carrier surfaces. Biofilm samples were centrifuged at 10,000 × g for 10 min and the resulting biomass pellets were used for genomic DNA extraction.^11^ For characterizing plant root microbiota over the course of a lettuce growth cycle, samples of the root entire system were collected from lettuce seedlings grown for two weeks in peat moss and also from lettuce roots grown for four and six weeks in the hydroponics beds. Samples were frozen in liquid nitrogen and ground into small pieces prior to DNA extraction.^36^

At the end of the aquaponics cycle, three fish from each system were selected and euthanized. Whole gut and gill samples were taken using sterile scalpel blades, air-dried in a laminar flow hood, and ground prior to DNA extraction.^37^ Samples were also collected from water and sponges in the mechanical filters at the end of operation. 4L water from each fish tank was filtered (0.2 micron), and filters were treated the same as biofilm carriers for DNA extraction.^11^

Genomic DNA from all samples were extracted using the FastDNA SPIN Kit for Soil (MP Biomedicals Inc.) along with blank controls. Quality and quantity of the extracted DNA were measured using Nanodrop (NanoDrop 2000 Spectrophotometer, Thermo Fisher Scientific) and a Qubit dsDNA HS Assay Kit, according to manufacturer’s protocol (Invitrogen Qubit Fluorometer, Thermo Fisher Scientific). The extracted DNA was tested for the presence of enzymatic inhibitors via PCR amplification of 16S rRNA genes (515F and 806R primers) prior to sequencing.

#### Sequencing and statistical analysis

The V3-V4 region of 16S rRNA was shown to be optimal for profiling microbial communities,^38^ with maximum phylogenetic coverage.^39^ We targeted the V3-V4 region of 16S rRNA genes of our samples and a control mock community for library preparation and sequencing by a commercial sequencing provider (Genome Quebec, Canada) using the Illumina MiSeq platform (MiSeq PE 250 bp). The unfiltered sequence data in this study was deposited in the NCBI Short Read Archive under accession number PRJNA941956.

The paired-end fastq files were processed with DADA2 pipeline^40^ version 1.16 as an R script (in R4.2.1). Filtering was performed with the FilterAndTrim function to keep the first 240 bases of the forward reads and 220 bases of the reverse reads. Error rates model learning [*(learnErrors)*] and ASV inference [*dada()*] were performed in R with the DADA2 default parameters. After clustering, chimeric sequences were identified and removed (Table S1). The Silva database (silva_nr_v138) was used to assign taxonomy to the resulting amplicon sequence variants (ASVs). Sequences assigned to chloroplast and mitochondria were removed. Accuracy of the inferred sequence variants was evaluated by comparing to the accuracy of the control mock community.

Sequences were rarified to the depth of the shallowest sample (18000 reads) using *rarefy_even_depth()* from phyloseq package. Metrics of dissimilarity for samples were calculated with the Bray-Curtis distance metric using *ordinate()* from phyloseq package. Nonmetric multidimensional scaling (NMDS) was used to visualize patterns of similarity among samples in two dimensions, using *plot_ordination()* from phyloseq package. Statistical support for differences between samples was calculated with the aid of permutational analyses of variance (PERMANOVA) using *adonis2()* function in the Vegan R package with Bonferroni corrections. A pairwise PERMANOVA was performed to examine pairwise differences in beta diversity using the R function *pairwiseAdonis()*, and p-values were corrected for multiple comparisons using Holm’s method. Chao1 and Shannon indices were used to assess alpha diversity of the samples. Significant shifts in the richness and diversity across sample groups were detected using ANOVA test and multiple comparisons by means of Tukey (*HSD.test()* function, p < 0.05). Analysis of Compositions of Microbiomes with Bias Correction 2 (ANCOM-BC2) was used for differential abundance analysis (DAA) with pH as a covariate to detect significant shifts in the rhizosphere microbiota in response to pH. Lastly, we used *ps_venn()* function from Russel88/MicEco package to diagram the shared ASVs across the aquaponics compartments in the four systems.

#### Metagenomic sequencing and analysis

To distinguish between *amoA* genes of AOB and comammox *Nitrospira*, high DNA samples extracted from the biofilm carriers of systems A2 and B2 were sequenced using the circular consensus sequencing (CCS) method. 500 ng of genomic DNA was used to make unamplified libraries using the SMRT-bell prep kit 3.0, according to the manufacturer’s recommendations. gDNA was sheared to a targeted fragment size of 12 kb using Megaruptor and Long Hydropores (Diagenode, Denville, NJ, USA). Sheared gDNA were concentrated using AMPure PB Beads according to the manufacturer recommendations (Pacific Biosciences, Menlo Park, CA, USA) and underwent two treatment procedures for DNA damage repair and end-repair. Barcoded overhang Hairpins adapters from the manufacturer were ligated to the fragment ends to create SMRT-bell templates used for sequencing. SMRT-bell templates were purified using an exonuclease procedure to remove any free ends molecules or no adapter templates. Libraries were sequenced on a PacBio RS II Single Molecule, Real-Time (SMRT.) DNA Sequencing System (Pacific Biosciences, CA, USA) using one SMRT cell 8M. High-fidelity reads were then generated with the “ccs” module within the SMRT link v10.0 package. For biofilm carriers A2 and B2, 190,192 and 97,669 number of reads, with mean length of 5905.3 bp and 6733.6 bp and Mean Quality (Q-score) of 58.4 and 56.1 were obtained, respectively.

Translation alignment of HiFi reads against protein database was performed using *DIAMOND* snakemake workflow (Portik et al., 2022). NCBI non-redundant protein database (NCBI-nr gz-db/FASTA-2023) was downloaded and indexed with *DIAMOND* prior to running the pipeline (diamond makedb –in nr.gz –db diamond_nr_db –threads 24). Default settings were used for configuring the analysis (chunks: 4, block_size: 12, threads: 24, hit_limit: --top 5, readassignmentmode: readCount, minSupportPercent: 0.01). DIAMOND workflow was run on a local system using long-read settings (--range-culling). This snakemake analysis identifies and removes CIGAR strings with illegal frameshift characters. sam2rma (-alg longReads) and rma2info are the executable tools required for this workflow. The resulting unfiltered RMA files were used to summarize the resulting alignment using MEGAN-CE with minimum threshold filtering for hits. Read counts and read-based profiling of the NCBI taxonomy were computed using the Long Read LCA algorithm. Also, the best-hit algorithm with SEED, eggNOG, and InterPro2GO was used for functional classification. The sequence reads that were initially aligned to pMMOs in the translated search were subsequently re-mapped to NCBI nucleotide database in order to determine the specific genus or species they were associated with.
