# Supplementary material for: Inoculum and pH effects on ammonium removal and microbial community dynamics in aquaponics systems
Source: iScience. 2024 Feb 1;27(3):109073. doi: 10.1016/j.isci.2024.109073 (PMC10867649; doi:10.1016/j.isci.2024.109073)
Supplement: Document S1. Figures S1–S11 and Tables S1 and S2 [file mmc1.pdf]

iScience, Volume *27*

Supplemental information

Inoculum and pH effects on ammonium removal and microbial community dynamics in aquaponics systems

Peyman Derikvand, Brittany Sauter, Andrew Keddie, and Lisa Y. Stein

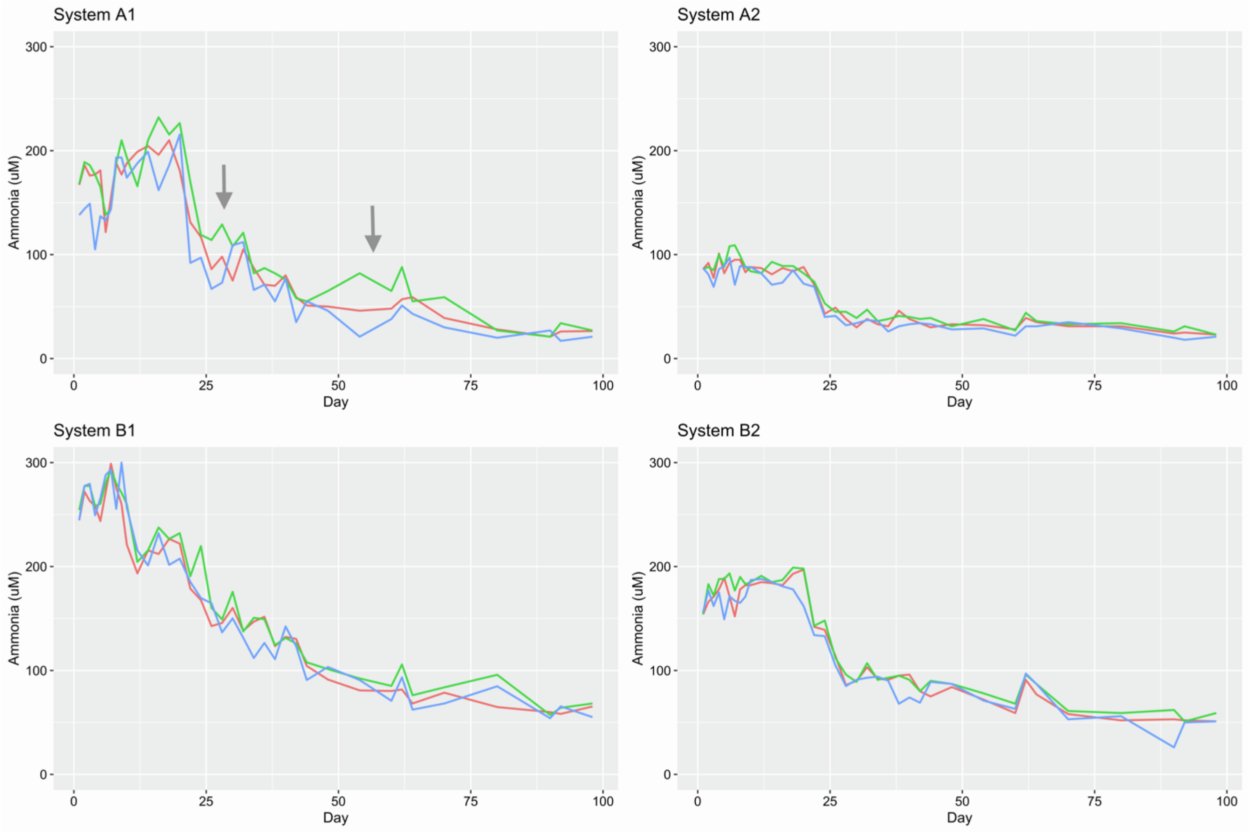

Fig S1. Variation of ammonia concentrations in four different aquaponics systems over three plant harvesting rounds. Related to Table 1. Arrows indicate time points where lettuce was harvested. Green: 30 min post-feeding, red: 2 h post-feeding, and blue: 5 h post-feeding.

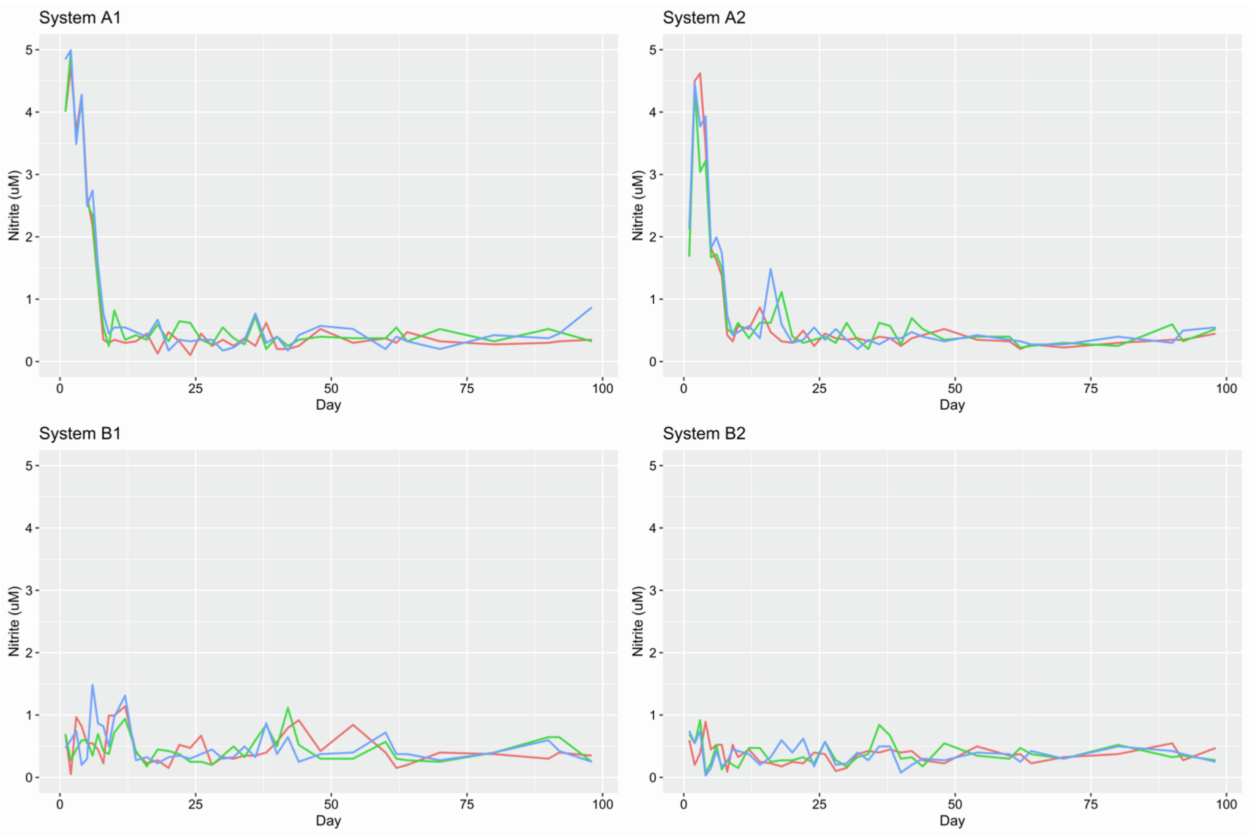

Fig. S2. Variation of nitrite concentrations in four different aquaponics systems over three plant harvesting rounds. Related to Table 1. Green: 30 min, red: 2 h, and blue: 5 h after feeding.

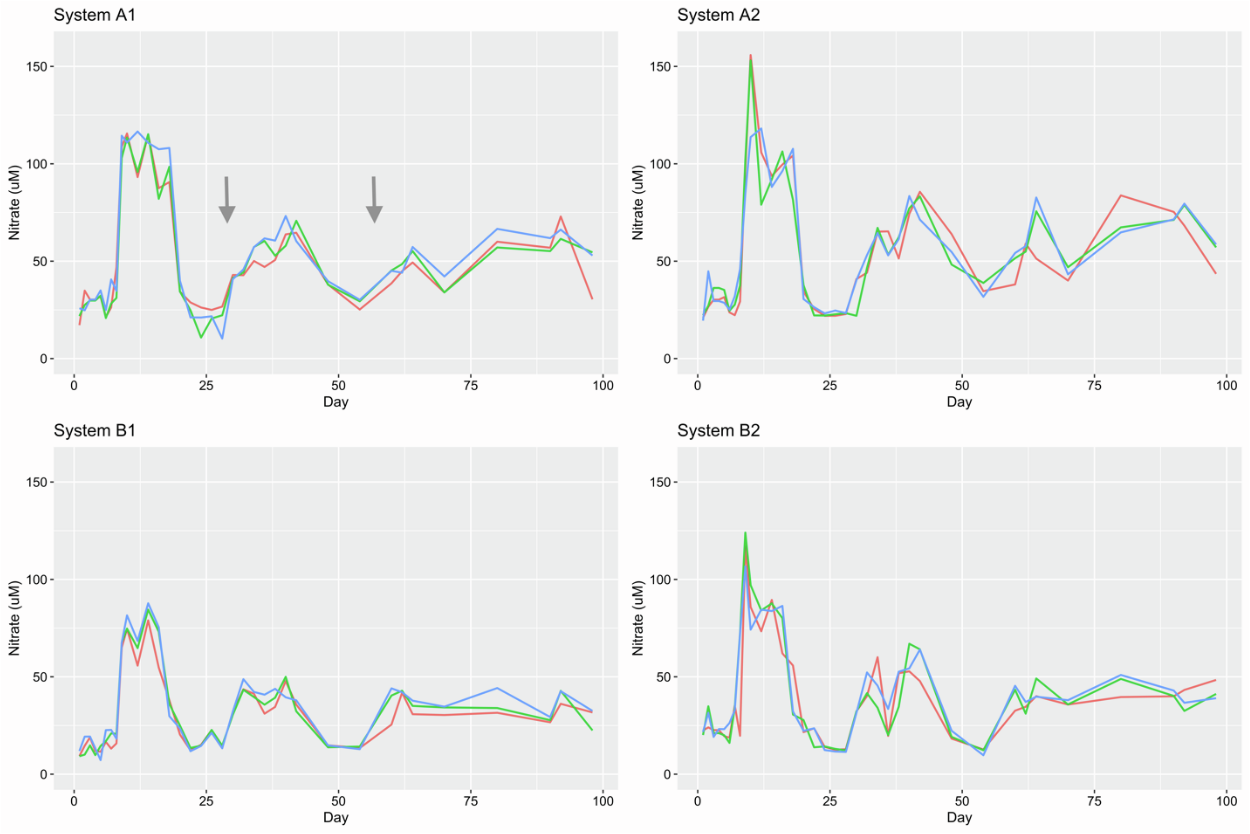

Fig. S3. Variation of nitrate concentrations in four different aquaponics systems over three plant harvesting rounds. Related to Table 1. Arrows on System A1 panel indicate harvesting time points and are the same for the other three panels. Green: 30 min, red: 2 h, and blue: 5 h after feeding.

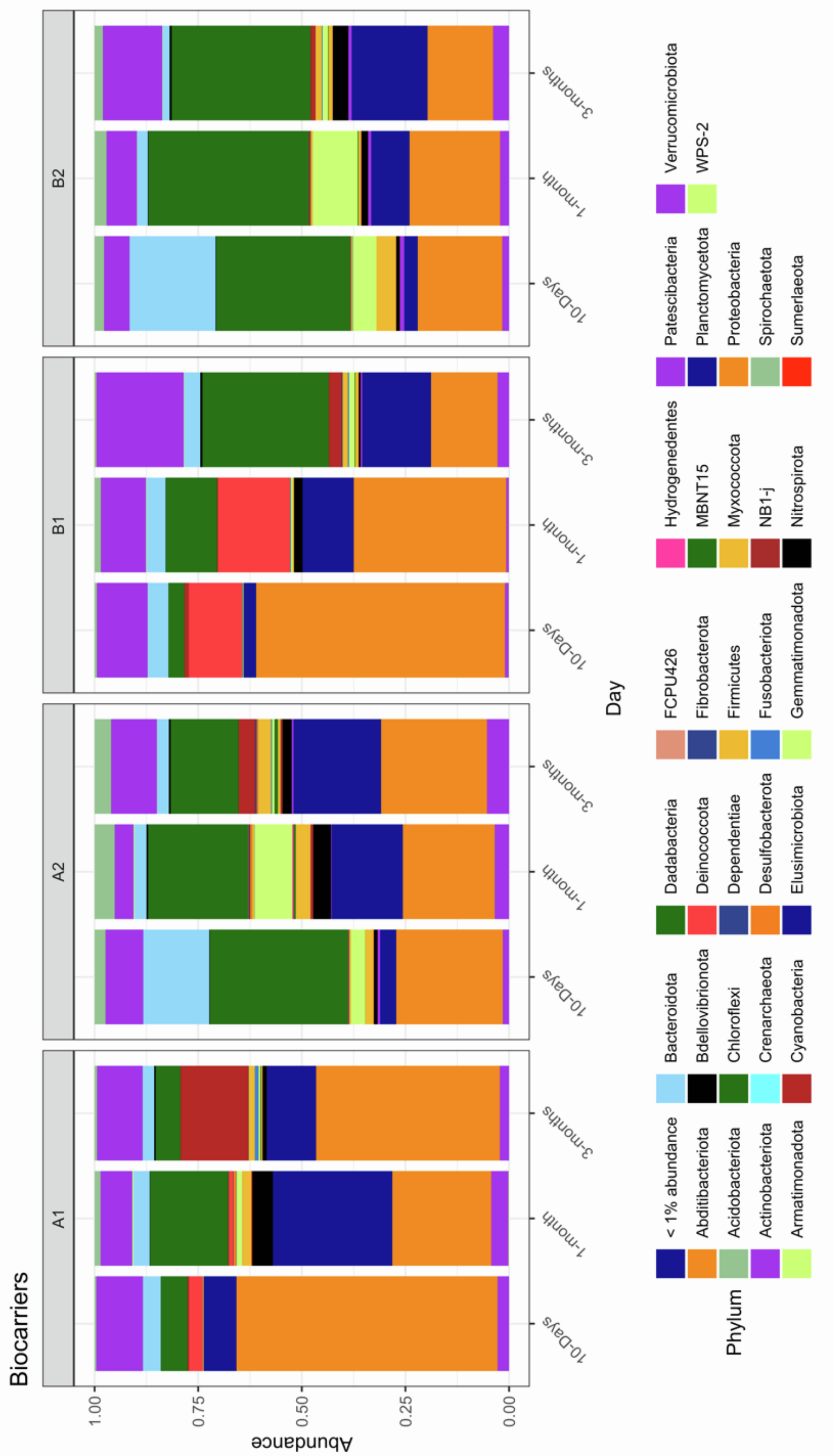

Fig. S4. Changes in microbial community compositions of biofilters (n=5 for each tank and each time point), after operation for 10 days, one month and three months based on ASV analysis. Related to Figure 2 and Table 2. ASVs were determined at the phylum level. Only phyla representing more than 1% of the total reads are presented.

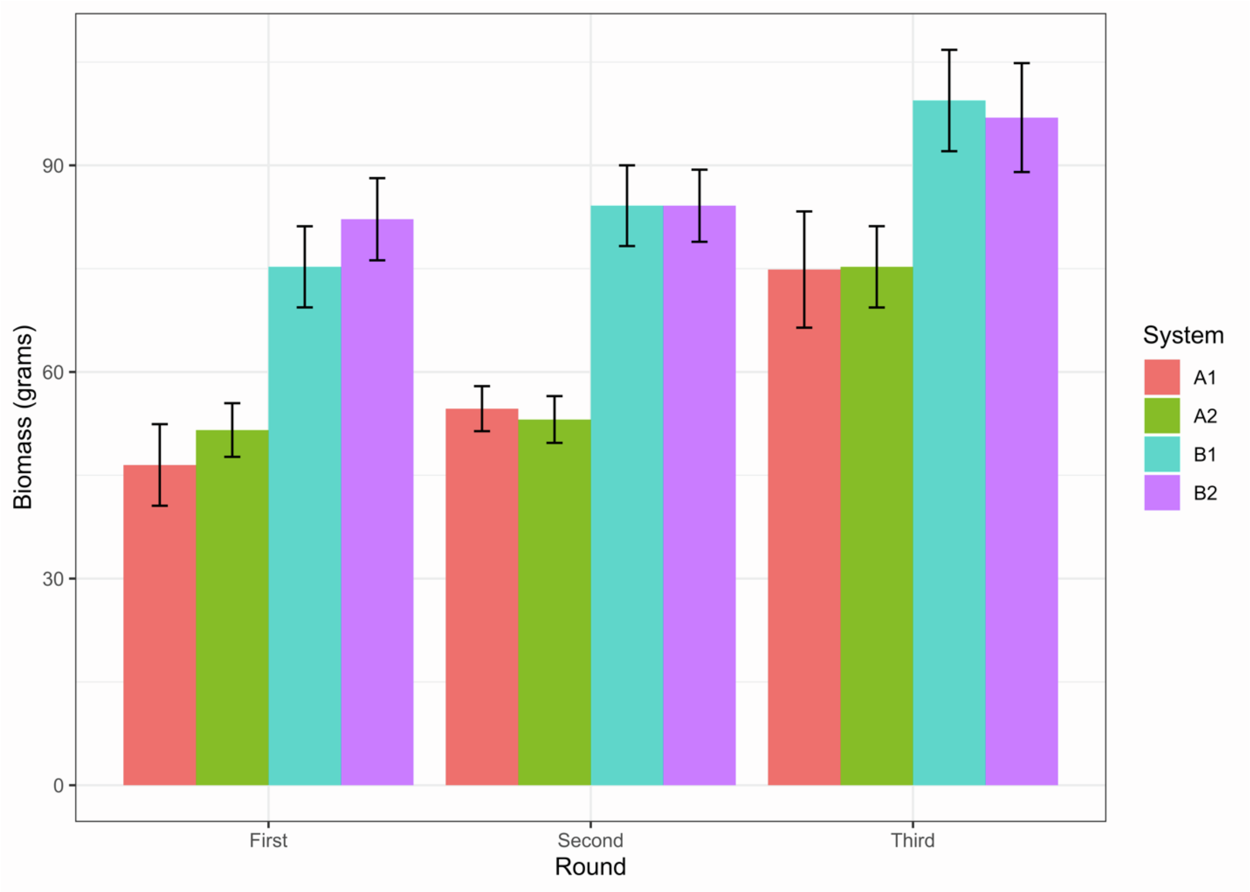

Fig. S5. Lettuce biomass weights at the end of three harvesting rounds from the four aquaponics systems. Related to Figure 2 and Table 2. The first and second rounds were harvested after 4 weeks, and the third round was harvested after six weeks. Standard deviations represent the variation in average weight of individual lettuce heads (n=14 per system)

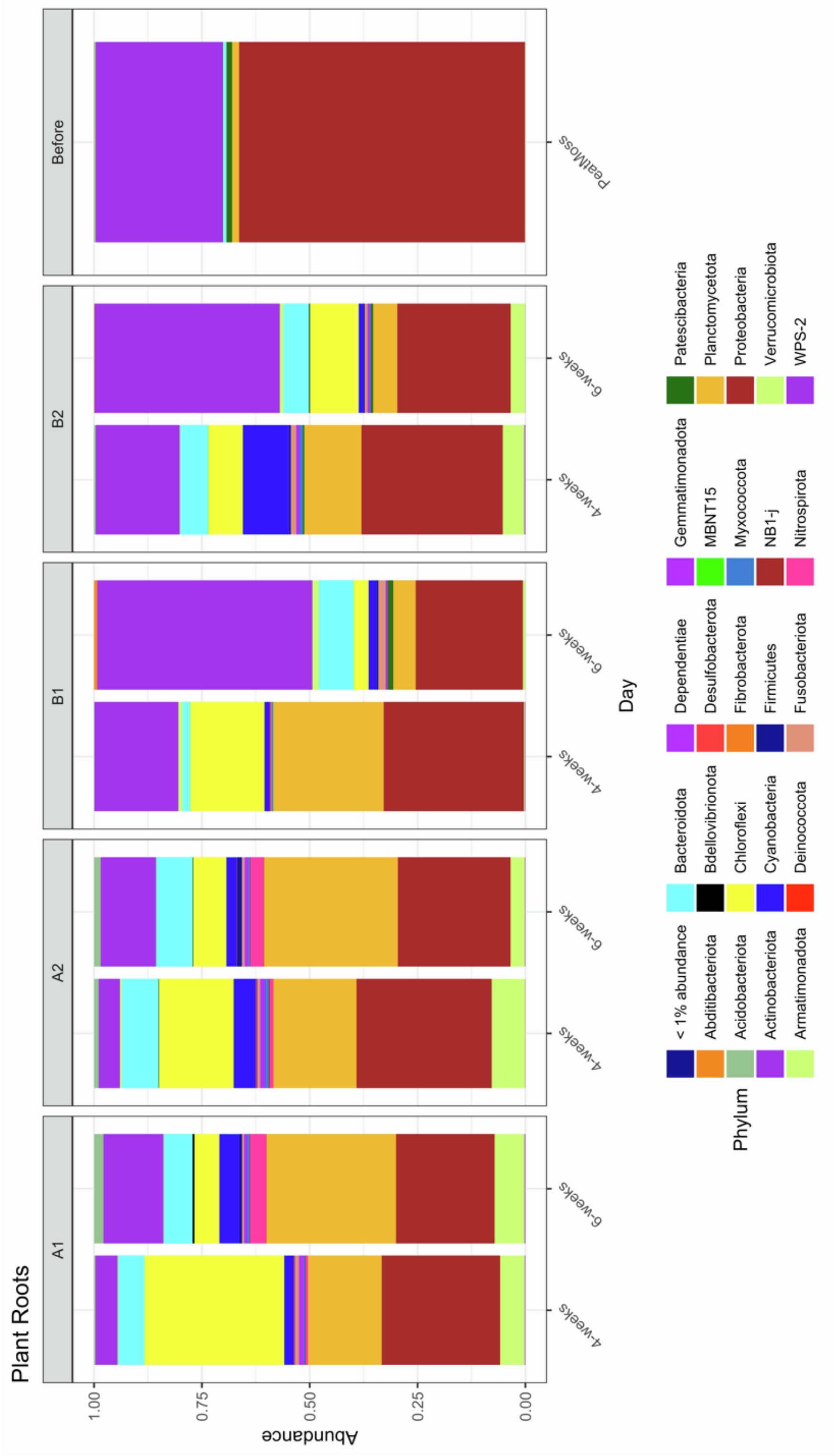

Fig. S6. Classification of ASVs derived from microbiome sequencing of lettuce root samples (n=3). Related to Table 2. Relative abundance of each phylum-level group is indicated as a percentage of the total population for each of the four aquaponics systems. Only ASV groups with greater than 1% sequence abundance are shown.

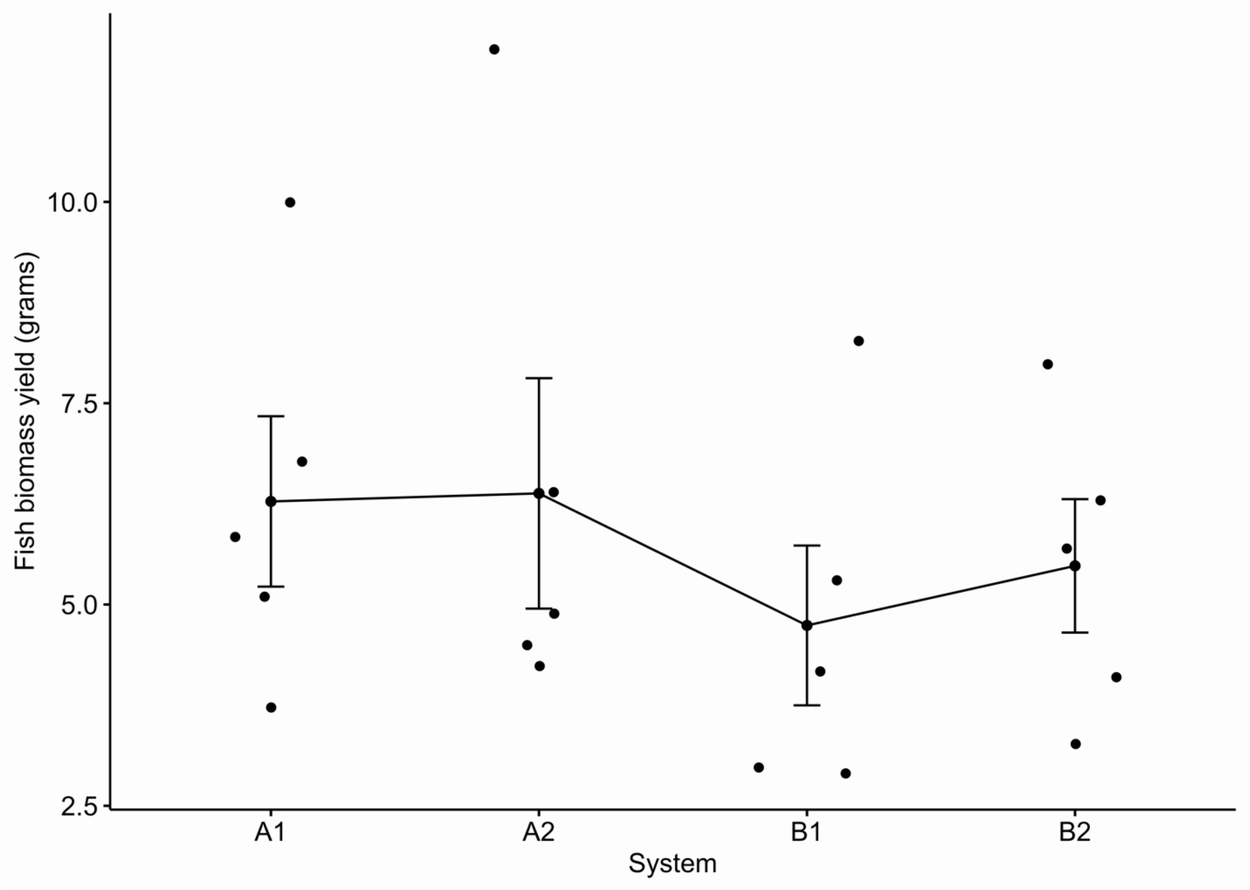

Fig. S7. Average yield of fish after 144 days of aquaponics systems operation for each system. Related to Figs. 1 & 2. The weight for each of 5 fish per tank are represented by dots, and the standard deviation is based on average fish weight per system.

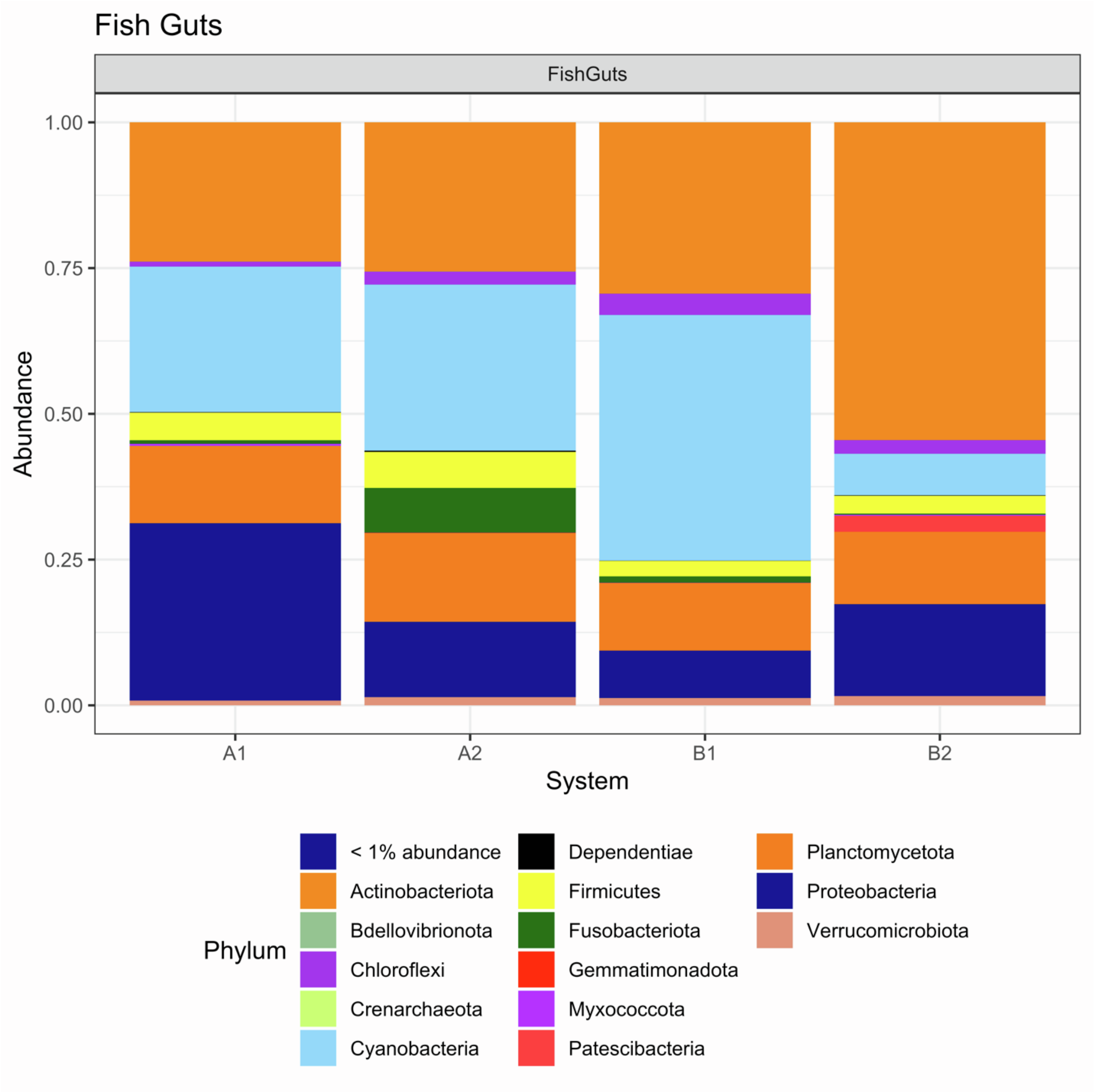

Fig. S8. Classification of ASVs derived from microbiome sequencing of fish gut samples (n=3) from each tank. Related to Figs. 1 & 2. Relative abundance of each phylum-level group is indicated as a percentage of the total population for each of the four aquaponics systems.

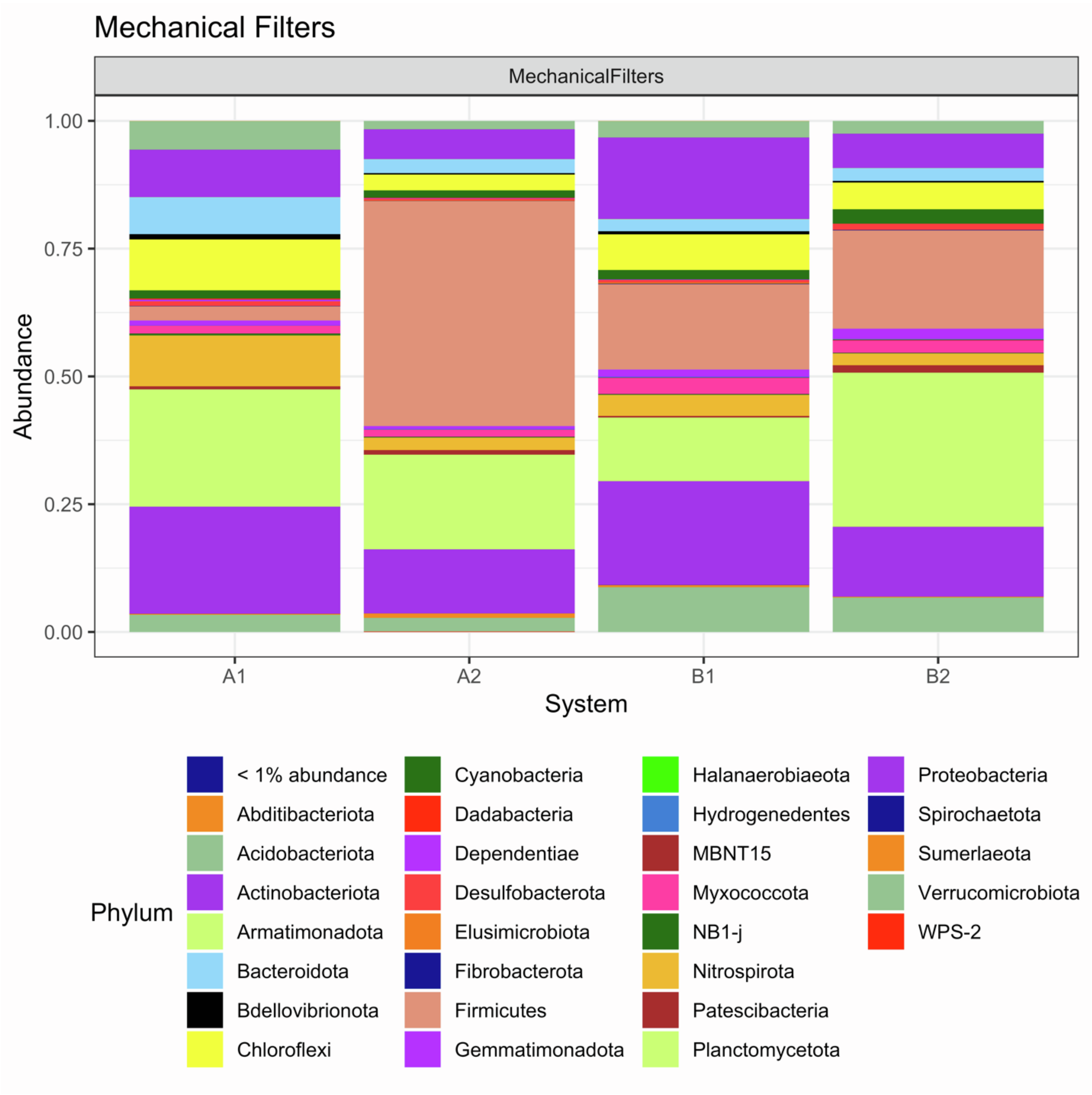

Fig. S9. Classification of ASVs derived from microbiome sequencing of samples from the mechanical filters (n=3) for each of the four aquaponics systems. Related to Figs. 1 & 2. Relative abundance of each group is indicated as a percentage of the total population.

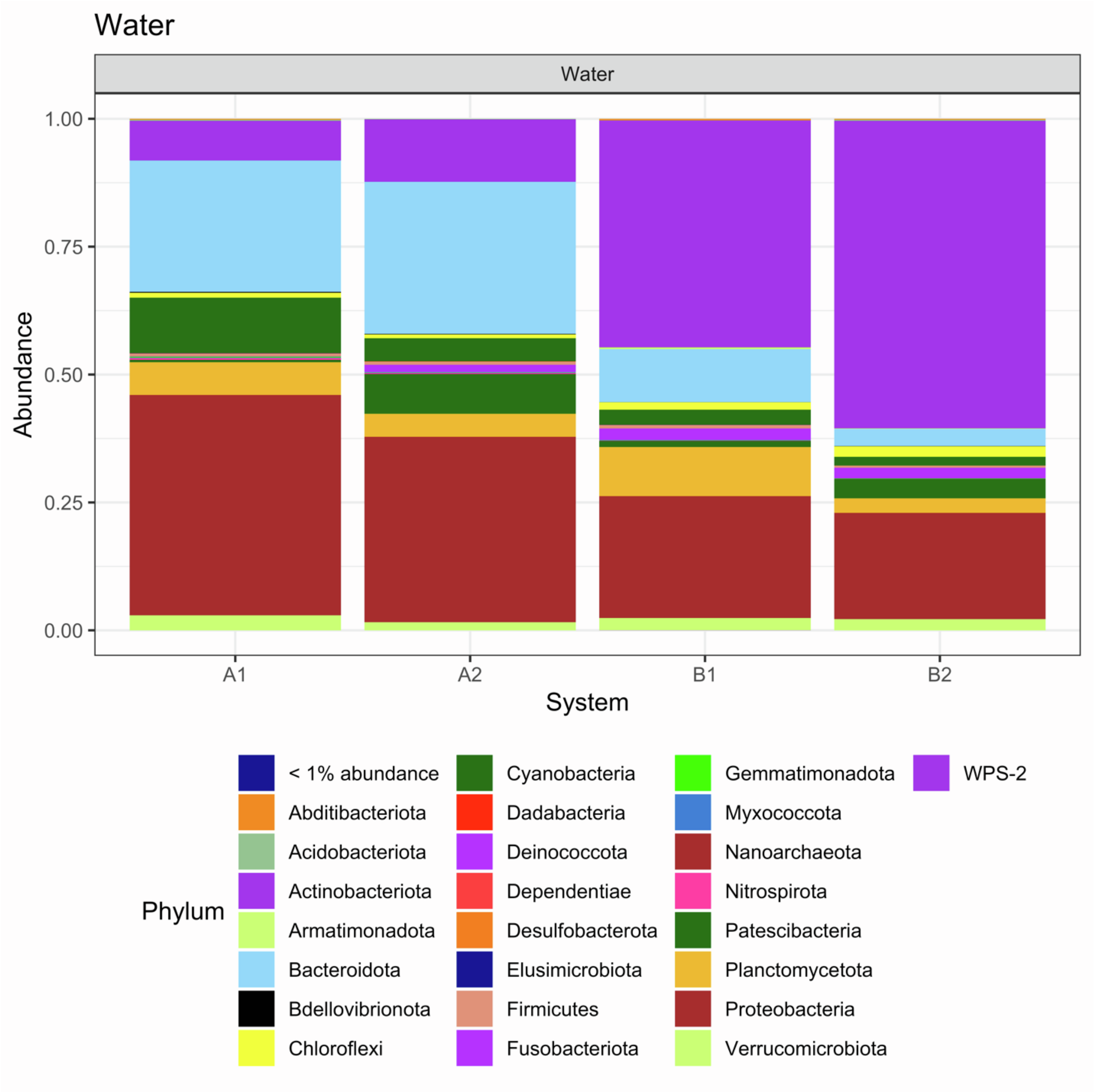

Fig. S10. Classification of ASVs derived from microbiome sequencing of water samples (n=3 for each tank). Related to Figs. 1 & 2. Relative abundance of each phylum-level group is indicated as a percentage of the total population for each of the four aquaponics systems.

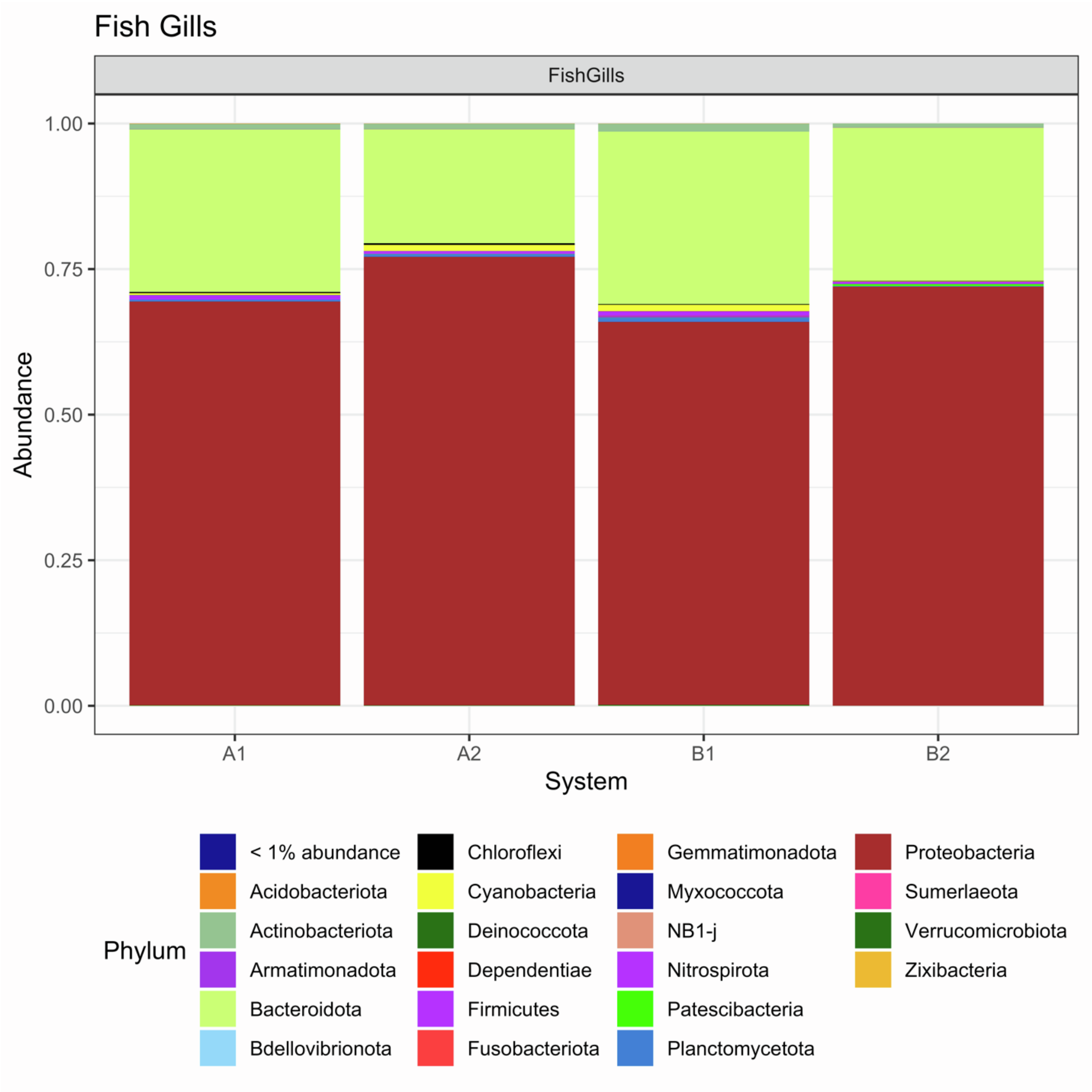

Fig. S11. Classification of ASVs derived from microbiome sequencing of fish gill samples (n=3 for each tank). Related to Figs. 1 & 2. Relative abundance of each phylum-level group is indicated as a percentage of the total population for each of the four aquaponics systems.

Supplemental Table 1. DADA2 filtering outputs. Related to STAR methods**.** The table shows the minimum and maximum number of reads per sample after the full DADA2 pipeline for a subset of samples.

Samples	input	filtered	denoisedF	denoisedR	merged	nonchim
Roots in peatmoss	49919	42416	38870	40529	33770	31638
Roots-4weeks-pH7.6	39405	33641	31565	32402	25309	24101
Roots-6weeks-pH7.6	47346	36138	32576	32718	31461	30792
Water-pH7.6	47120	41773	40722	40952	35179	32336
Biofilters-Day10-pH7.6	41305	34859	33282	33817	28913	25719
Biofilters-1month-pH7.6	51023	42499	39409	39240	36718	33463
Biofilters-3months-pH7.6	46711	41288	40116	39571	38771	35698
Fish gut-pH7.6	46060	39862	38553	38898	33368	32091
Fish gills-pH7.6	38498	33492	29608	32082	28294	26280
Mechanical filter-pH7.6	48473	42890	40617	41328	32532	30618

Supplemental Table 2. Analysis of Compositions of Microbiomes with Bias Correction 2 (ANCOM-BC2). Related to Table 2. Differential abundance analysis (DAA) was conducted with pH as a covariate to detect significant shifts in the rhizosphere microbiota at the Family level in response to pH.

Family	Log-fold change pH7.6 – pH6	Adjusted P-value
Micrococcales_Microbacteriaceae	-4.08	0.00001
Corynebacteriales_Mycobacteriaceae	-2.31	0.00110
Micromonosporales_Micromonosporaceae	-4.01	0.00001
Xanthomonadales_Rhodanobacteraceae	-2.47	0.00991
Nitrospirales_Nitrospiraceae	3.92	0.00001
Actinobacteria_PeM15_NA	-3.65	0.00002
Verrucomicrobiales_ Verrucomicrobiaceae	3.56	0.00003
Vicinamibacterales_Vicinamibacteraceae	2.81	0.00022
Kineosporiales_Kineosporiaceae	-3.64	0.00002
Armatimonadales_NA	-2.50	0.00289
Cellvibrionales_Cellvibrionaceae	2.25	0.01622
Isosphaerales_Isosphaeraceae	-2.60	0.04303
Acidimicrobiia_Microtrichales_NA	-2.31	0.02843
Acidobacteriota_Blastocatellia_11-24_NA	-2.22	0.01164
